# Positive hysteresis in emotion recognition: Face processing visual regions are involved in perceptual persistence, which mediates interactions between anterior insula and medial prefrontal cortex

**DOI:** 10.3758/s13415-022-01024-w

**Published:** 2022-07-20

**Authors:** Andreia Verdade, Teresa Sousa, João Castelhano, Miguel Castelo-Branco

**Affiliations:** 1 Coimbra Institute for Biomedical Imaging and Translational Research (CIBIT), University of Coimbra, Coimbra, Portugal; 2 Institute of Nuclear Sciences Applied to Health (ICNAS), University of Coimbra, Coimbra, Portugal; 3 Faculty of Medicine, University of Coimbra, Coimbra, Portugal

**Keywords:** Decision-making, Dynamic facial expression, Emotion perception, fMRI, Perceptual history

## Abstract

Facial emotion perception can be studied from the point of view of dynamic systems whose output may depend not only on current input but also on prior history — a phenomenon known as hysteresis. In cognitive neuroscience, hysteresis has been described as positive (perceptual persistence) or negative (fatigue of current percept) depending on whether perceptual switching occurs later or earlier than actual physical stimulus changes. However, its neural correlates remain elusive. We used dynamic transitions between emotional expressions and combined behavioral assessment with functional magnetic resonance imaging (fMRI) to investigate the underlying circuitry of perceptual hysteresis in facial emotion recognition. Our findings revealed the involvement of face-selective visual areas — fusiform face area (FFA) and superior temporal sulcus (STS) — in perceptual persistence as well as the right anterior insula. Moreover, functional connectivity analyses revealed an interplay between the right anterior insula and medial prefrontal cortex, which showed to be dependent on the presence of positive hysteresis. Our results support the hypothesis that high-order regions are involved in perceptual stabilization and decision during perceptual persistence (positive hysteresis) and add evidence to the role of the anterior insula as a hub of sensory information in perceptual decision-making.

**Supplementary Information:**

The online version contains supplementary material available at 10.3758/s13415-022-01024-w.

## Introduction

A dynamic system’s output can be determined not only by the external inputs but also by its history (Luenberger, 1979). When the output of a system depends on the direction of change of an input control parameter, we observe hysteresis (Warburg, 1881). The phenomenon, widely studied in magnetism (Jiles & Atherton, 1986; Stoner & Wohlfarth, 1991; Warburg, 1881), has gained attention in the field of cognitive neuroscience, particularly in perception (Kleinschmidt et al., 2002; Kobayashi & Hara, 1993; Williams et al., 1986). Accordingly, perceptual hysteresis is defined to occur when the transition point between two perceptual states is dependent on the direction of the visual input change (Dubé, 1997). This history-based dependence plays a role in the disambiguation of multistable ambiguous stimuli (Hock & Schöner, 2011; Kleinschmidt et al., 2002; Sacharin et al., 2012; Witthoft et al., 2018).

The phenomenon has been described in terms of two types of behavior depending on the temporal context: positive and negative hysteresis. Positive perceptual hysteresis occurs when perception persists on the original percept even though the actual physical changes of the stimulus favor an alternative percept (Hsu & Wu, 2019; Kleinschmidt et al., 2002; Pearson & Brascamp, 2008). If, however, the perceptual switch takes place earlier, then negative hysteresis occurs. These two signatures have been reported to reflect the underlying perceptual mechanisms’ contribution to stabilizing the current percept (Hock & Schöner, 2011; Kleinschmidt et al., 2002; Sacharin et al., 2012; Witthoft et al., 2018). Positive hysteresis has been related to memory (persistence) mechanisms due to recent perceptual experience (Hock & Schöner, 2011; Kleinschmidt et al., 2002; Liaci et al., 2018; Sacharin et al., 2012; Sayal et al., 2020; Verdade et al., 2020); while negative hysteresis has been related to adaptation/habituation mechanisms that lead to earlier switches (Liaci et al., 2018; Pisarchik et al., 2014; Sayal et al., 2020; Webster et al., 2004). Recent research has suggested that the interplay between these two mechanisms ultimately determines the perceptual hysteresis effect (Liaci et al., 2018; Sayal et al., 2020; Schwiedrzik et al., 2014). Whether these mechanisms arise from the same neural process (Blake et al., 2003; Chen & He, 2004; Gepshtein & Kubovy, 2005; Orbach et al., 1963) or map into anatomically and hierarchically distinct networks (Fritsche et al., 2017; Lopresti-Goodman et al., 2013; Sayal et al., 2020; Schwiedrzik et al., 2014) remains under debate. However, it appears to be a consensus that the phenomenon of perceptual hysteresis reflects the contribution of top-down signals in the disambiguation of ambiguous stimuli (Fritsche et al., 2017; Kleinschmidt et al., 2002; Liaci et al., 2018; Liberman et al., 2018; Mei et al., 2019; Sayal et al., 2020; Verdade et al., 2020).

Several high-order brain regions have been reported to underlie hysteresis in perception, such as the intraparietal sulcus, the dorsomedial prefrontal cortex, the supplementary motor area, and the anterior insula (Sayal et al., 2020; Schwiedrzik et al., 2014). However, their role in perceptual maintenance and interplay with low-order regions remains unclear. For example, it has been proposed that early visual areas, such as the face fusiform area (FFA), may be relevant for perceptual maintenance (Gazzaley et al., 2004), which may be directly related to positive hysteresis. Moreover, in which concerns facial emotion expressions, the posterior superior temporal sulcus (pSTS) may have a relevant role in this context (Direito et al., 2019).

The anterior insula is one of the core hubs of the salience network, whose function involves perceiving and responding to homeostatic demands (Menon, 2015; Menon & Uddin, 2010; Seeley, 2019; Uddin et al., 2017). The region functions as an integral node, integrating autonomic, visceral, and sensory information while mediating the dynamic switching between the central executive and the default mode networks to generate the appropriate response to salient stimuli. The mediating role of the insula in the recruitment of the central executive network is of particular importance for perceptual decision-making (Craig, 2009; Singer et al., 2009; Xue et al., 2010, Castelhano et al., 2014). In fact, several neuroimaging studies have reported the anterior insula to play a critical role in perceptual decision processes, with a positive correlation between greater activations and perceptual difficulty (Chand & Dhamala, 2016; Lamichhane et al., 2016; Pessoa & Padmala, 2005; Thielscher & Pessoa, 2007). However, the relative role of visual and high-order regions, such as the insula, in perceptual decision remain poorly understood, and hysteresis provides a paradigm to tackle their relative contributions.

We sought to study the underlying neural mechanisms of perceptual hysteresis in the context of emotion recognition. Hysteresis in emotion perception was described first by Kobayashi and Hara (1993). The authors demonstrated that when seeing facial expressions changing dynamically, the perception was dependent on the direction of the change. However, research on emotional facial expressions perception has, in general, been overlooked in this context. First, in line with the basic theory of emotions, the majority of studies have used static stimuli, disregarding important aspects of the dynamic nature of emotions emerging in everyday life interactions (Dubé, 1997; Kamachi et al., 2013; LaBar et al., 2003; Sacharin et al., 2012; Sato et al., 2004; Trautmann et al., 2009; Trautmann-Lengsfeld et al., 2013). Second, more recent studies using dynamic emotional facial expressions have disregarded transitions between emotional states, limiting their research by focusing on changes from and to neutral expression (Kamachi et al., 2013; LaBar et al., 2003; Sato et al., 2004; Webster et al., 2004). Moreover, even those have not explored temporal context effects on the current perception (LaBar et al., 2003; Webster et al., 2004).

In a previous study, we established that the perception of reality-based changing emotion expressions was dependent on recent perceptual history and its direction of variation (Verdade et al., 2020). The basic findings were that positive hysteresis dominated in such percepts, and positive emotions dominated in the temporal history effects. In the current study, we used realistic stimuli based on dynamic transitions of emotional expressions to investigate the neural correlates of perceptual hysteresis. We hypothesized that early visual areas are a substrate of perceptual persistence, and the anterior insula is a critical region in the network to which perceptual hysteresis may map into in the context of emotion recognition. In particular, we wanted to test the role of the anterior insula in perceptual hysteresis and its contribution to overall perception, as previously postulated (Sayal et al., 2020; Schwiedrzik et al., 2014). Therefore, we sought to investigate its role in integrating sensory information and higher-order cognitive networks in decision-making (Menon, 2015; Menon & Uddin, 2010). We were mostly interested in the right hemisphere because of previous neuroimaging and neurophysiology evidence of lateralized face processing and emotion (De Winter et al., 2015), as well as insula lateralization in emotion perception (Zhang et al., 2018) and perceptual hysteresis (Sayal et al., 2020). Different pairs of emotions were used to create the dynamic transitions and study the neural correlates of recent perceptual experience effects on perception while comparing to the case of no perceptual influence.

## Materials and methods

### Participants

Seventeen healthy young adults participated in this experiment (9 females, mean age 27.53 ± 4.02 years). All had a normal or corrected-to-normal vision and no history of neurological or psychiatric diseases. Participants provided written, informed consent to take part in the study, following protocols approved by the Ethics Committee of the Faculty of Medicine of the University of Coimbra, in accordance with the Declaration of Helsinki.

### Experimental setup and apparatus

The data acquisition session comprised one structural magnetic resonance imaging (MRI) and six functional MRI (fMRI) runs (three dynamic transitions runs, and three static control runs). The dynamic transitions runs were aimed to access the contribution of recent perceptual experience to perception, while the static control runs established a control case of no perceptual influence on the current perception.

The order of the total sum of six runs was random for each subject. Presentation software (version 20.1, Neurobehavioral Systems, Inc., Albany, CA) was used to design and present the stimulus of both tasks and to collect participants’ responses. Stimuli were presented in the centre of an LCD screen with a refresh rate of 60 Hz and a resolution of 1920 x 1080 pixels and participants were positioned at 156 cm from the display screen. Participants’ reports were recorded using a fibre-optical MR-compatible response box (Cedrus Lumina LSC-400B).

### Stimuli

Frontal-view images of posed expressions of three basic emotions—sadness, happiness, and anger—and respective neutral expressions of four females and three males were chosen from the Extended Cohn-Kanade Dataset (Lucey et al., 2010). The images were cropped by a uniform rectangle allowing the preservation of the internal features of the faces and also the surrounding external features (visual angle of 5.05° horizontally and 4.03° vertically) and corrected for luminance levels (average luminance value of 44.76 ± 1.73 cd/m^2^). All stimuli were presented on a grey background (23.10 cd/m^2^).

**Fig. 1:**
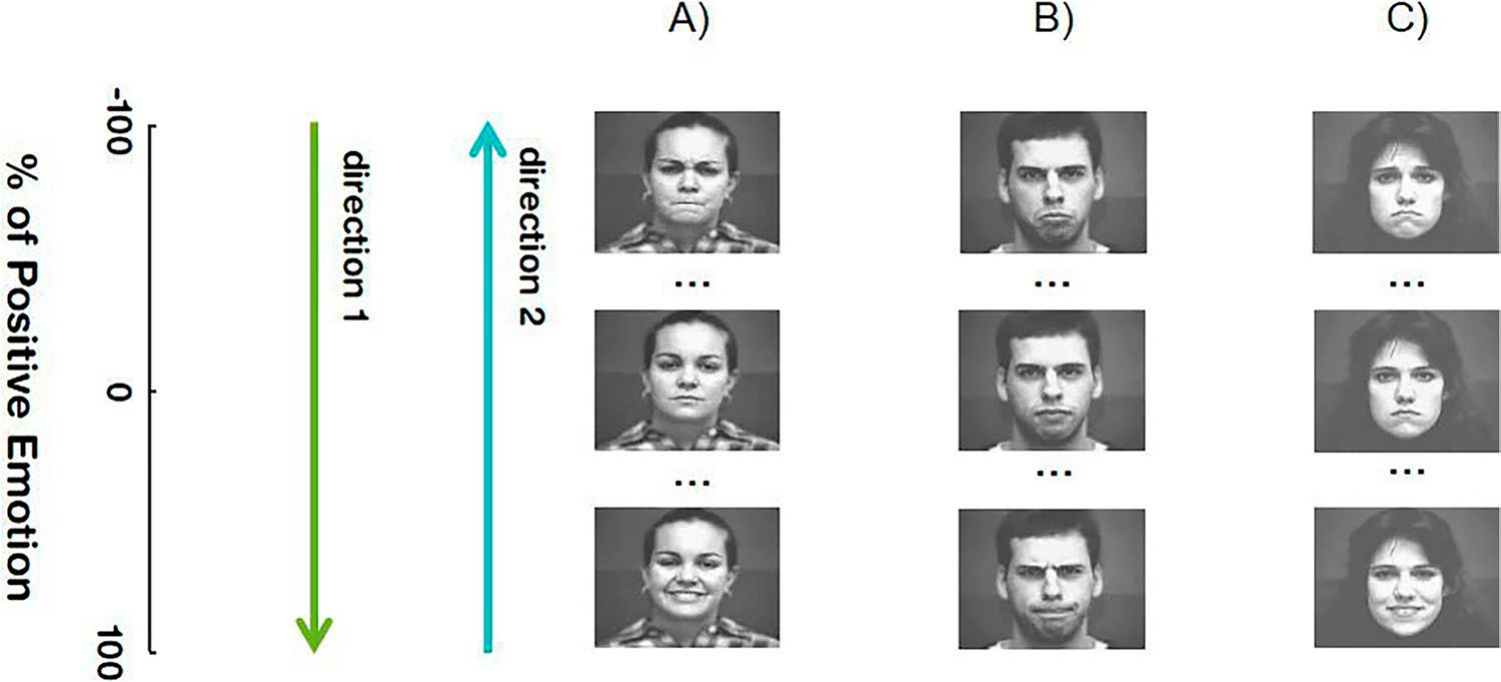
**Examples of dynamic transitions used during visual stimulation.** Each stimulation trial consisted in a dynamic transition between two emotion types (direction 1 and direction 2), always passing through a neutral expression. The source emotion expression gradually evolved to a neutral expression, which subsequently evolved to a target emotion expression. The neutral expression corresponded to the zero percent of the positive (when this was the case) emotion (used to parametrize each morphing sequence). The examples illustrate transitions between anger and happiness (***a***), sadness and anger (***b***), and between sadness and happiness (***c***), which were randomly chosen from the dataset. Facial images are from the Extended Cohn-Kanade Dataset (Lucey et al., 2010)

Dynamic transitions consisted of a *source* emotion expression (E1) that gradually evolved to a *neutral* expression (N) and then into a *target* emotion expression (E2), matching our previous study (Verdade et al., 2020). *Source* and *target* emotions were always different (Fig. 1 for some examples). The transitions were obtained by morphing sequences of digital images generated using MorphMan 4.0 software (STOIK, Moscow, Russia) and by applying approximately 80 fiducial markers, which were densely placed in face-relevant areas, such as the eyes, mouth, and corrugator muscles (Ekman & Friesen, 1978). Morphs were chosen to allow finer control over the rate and duration of the changing expressions, as previously done by others (LaBar et al., 2003; Sacharin et al., 2012; Sato et al., 2004; Sato et al., 2010; Webster et al., 2004).

In total, each dynamic emotion transition consisted of an array of 81 sequential images of the same actor with a morphing step of 2.50% (40 intermediate images between each emotion and neutral expression). The image sequences were converted into movie clips with a frame rate of 8.89 fr/s using MATLAB (R2019a, The MathWorks), totaling a morphing duration of 9 seconds. Each frame was presented for 113.92 milliseconds, except for the first and last morph frames, corresponding to the *source* and *target* emotion expressions, which remained on the screen for 500 milliseconds. Thus, each dynamic transition trial was in total 10 seconds long.^1^ More details on the stimulus properties and a complete description of tasks are provided below.

### Emotion recognition based on dynamic transitions

Each dynamic transition run included four dynamic transition trials of three emotion pairs in two stimulation directions (24 trials per run). The participants were asked to report the start and end of what they perceived as neutral within all trials via button press. Moreover, within each run, control dynamic transitions (from neutral to neutral) were presented in an interleaved fashion to be used as a baseline for the transitions between different expressions.

The dynamic transitions were based on three pairs of emotions: anger-happiness, sadness-anger, and sadness-happiness following two stimulation directions. Direction 1 — the *source* was the first emotion of the above-mentioned pairs, and the *target* was the second; direction 2—the *source* and the *target* were reversed (Fig. 1). It is important to take into account that the two basic emotions sadness and anger are located in the negative part of the valence axis in the two-dimensional space of valence and arousal (Russell, 1980) in contrast with the other emotion pairs. In the control dynamic transitions, used as a control for the changing facial expression itself, both the *source* and *target* images were also neutral expressions (distinct neutral expressions of an actor available in the used dataset). Thus, although the morphing and presentation parameters were similar to the remaining transitions, no emotional changes occurred.

**Fig. 2:**
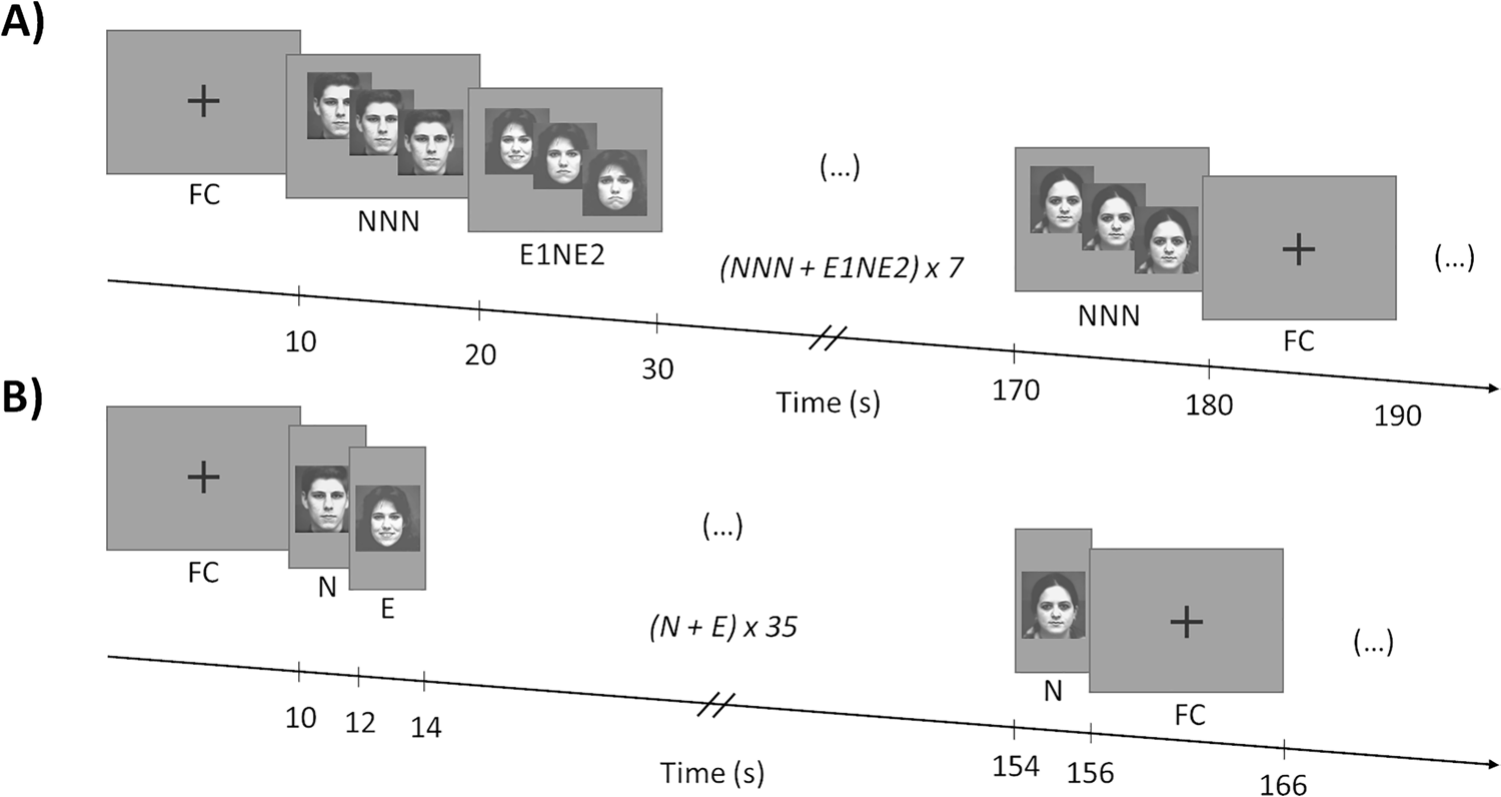
**Summary of the experimental design used in our study**. ***a*** Each dynamic transition run included four dynamic transition trials of each pair of emotions in both directions of stimulation. Trials were presented in a pseudo-randomized order to ensure that there were no two consecutive dynamic transitions of the same actor. Each run included three sequences of eighth dynamic transitions (E1NE2 or E2NE1), interleaved with control transitions (neutral to neutral [NNN]), and starting with a fixation cross (FC). Each condition lasted for ten seconds. The FC was additionally shown at the beginning and end of each run. Participants were asked to press a response button whenever they detected a change to and fro*m a neutral facial expression.* ***b*** Each static control run included twelve repetitions of the nine static images taken from the dynamic transitions between one pair of emotions (E), interleaved with a neutral condition (N), each lasting two seconds. Each run began and ended with an FC of 10 seconds, which was also presented at the beginning of each sequence of 36 repetitions of the conditions of interest (E+N). The participants were instructed to classify the emotion perceived in each static image presented as a positive, neutral, or negative expression. Facial images are from the Extended Cohn-Kanade Dataset (Lucey et al., 2010)

Trials were presented in a pseudo-randomized order to ensure that there were no two consecutive dynamic transitions of the same actor. We ran three sequences of eighth dynamic transitions (emotion 1 to emotion 2), interleaved with control transitions (neutral to neutral), and starting with a black fixation cross (with a visual angle of 1.01° horizontally and 1.01° vertically) presented on a grey background. Each condition lasted for ten seconds. The fixation cross was additionally shown at the beginning and end of each run. An example of a stimuli sequence is shown in Fig. 2a.

Participants were informed of the presence of different types of dynamic transitions. Moreover, they were instructed to identify the neutral expression interval of each dynamic transition (source to target emotion transitions). They were asked to press the response button whenever they detected a change to and from a neutral facial expression (P1 and P2 moments reflecting emotion discrimination), thus identifying the beginning and offset of the interval of a neutral expression. Each run lasted 9.3 minutes. Participants performed one training session before starting the main experiment.

### Emotion recognition based on static facial expressions

To estimate the perceptual switch in the case of no recent perceptual history, the percentage of neutral report given for static images taken from the dynamic transitions between each pair of emotions, was evaluated. From each morphing sequence used for the dynamic transitions, nine relevant images were selected (Table 1) and randomly presented for 2,000 milliseconds. Because the facial snapshots were not sequentially organized, no perceptual bias due to recent experience was expected.

**Table 1:** Frames consisting of static facial expressions selected from the complete sequence used for the dynamic transitions. The correspondent percentage of negative (E1), positive (E2), and neutral emotion (N) are here summarized

Frame	Emotion percentage
1	E1 – 100%; N – 0%
9	E1 – 80%; N – 20%
17	E1 – 60%; N – 40%
33	E1 – 20%; N – 80%
41	E1 – 0%; N – 100%
49	N – 80%; E2 – 20%
65	N – 40%; E2 – 60%
73	N – 20%; E2 – 80%
81	N – 0%; E2 – 100%

To avoid confusing participants with images that could belong to transitions of different emotion pairs but have similar features, each run contained only images selected from one pair: static control run 1 — *anger* and *happiness*, static control run 2 — *sadness* and *anger*, and static control run 3 — *sadness* and *happiness*. Per run, there were 12 repetitions of the 9 static images taken from the dynamic transitions between 1 pair of emotions (108 trials), which were interleaved with a neutral condition. The neutral corresponded to one of the images chosen randomly from the control dynamic transitions (transitions between neutral expressions) and lasted also for 2,000 milliseconds. Considering that in such transitions, although morphing was occurring, there was no variation in emotion percentage, any frame would correspond to 100% neutral.

Each static control run began and ended with a black fixation cross (with a visual angle of 1.01° horizontally and 1.01° vertically) presented for 10 seconds on a grey background. It also was presented at the beginning of each sequence of 36 repetitions of the conditions of interest allowing participants to rest from the task (Fig. 2b). The participants were instructed to classify the emotion perceived in each static image presented as a positive emotional expression (E+), a neutral expression (N) or as a negative emotional expression (E-). This strategy allowed us generalization because the task becomes similar across conditions. For the more ambiguous case of the anger/sadness pair the ground truth for this assignment was decided by the participant, with a relative proportion of 10/17 considering it as the most positive emotion.

Importantly, even though the report approach is not exactly the same as the dynamic runs, it allowed us to establish which frames were perceived as neutral. Subsequently, we averaged the position of the earlier and latter frame participants reported as neutral expression as an approximation to the perceptual switch between the pair of emotions in the absence of perceptual history. Participants' responses were collected via a button press and each classification was assigned to a specific response button in the response box. Each stimulation run lasted 8.3 minutes.

### fMRI data acquisition

The structural and functional data were collected with a 3-Tesla Siemens Magnetom Tim Trio scanner equipped with a 64-channel head coil at the Institute of Nuclear Sciences Applied to Health (ICNAS), Coimbra, Portugal. The scanning session started with the acquisition of one T1-weighted Magnetization-Prepared Rapid Gradient-Echo (MPRAGE) sequence (TR ​= ​2,530 ​ms, echo time (TE) ​= ​3.5 ​ms, flip angle ​= ​7°, 192 slices, voxel size = 1.0 ​× ​1.0 ​× ​1.0 ​mm, field of view (FOV) ​= ​256 ​× ​256 ​mm). Six functional runs were then acquired using a T2∗-weighted gradient echo-planar imaging (EPI) sequence. These consisted of 280 ​volumes in the dynamic transitions runs and 249 ​volumes in the static control runs (TR ​= ​2,000 ​ms, TE ​= ​30 ​ms, flip angle ​= ​75°, 37 slices, voxel size = 3.0 ​× ​3.0 ​× ​3.0 ​mm, FOV = 210 ​× ​210 ​mm). In total, the scanning session lasted approximately 60 ​min.

### Metric of perceptual hysteresis

To investigate perceptual hysteresis, we compared the perceptual switch curves, estimated based on participants’ reports, in each direction of stimulation with the case of no recent perceptual influence (no perceptual history), for each pair.

Because in dynamic transitions participants were required to identify the first and last time points in which they perceived the neutral expression, this allowed us to define an interval of what they had perceived as a neutral expression. Trials in which only one or no response was given were excluded from the analysis due to the impossibility of defining the neutral interval (4.65% of the trials). The average point of each border of the neutral interval was used as the transition point between each pair of emotions for each stimulation trajectory of the dynamic runs. To estimate the perceptual switch in the case of no history, the percentage of the neutral report given in each static image shown in the static control runs was obtained, which allowed us to establish the frames perceived as neutral. Then, the average position of the earlier and latter frames reported by participants as neutral was calculated.

The difference between the absolute value of the perceptual switch to the alternative percept in each direction and the absolute value of the perceptual switch in the case of no history was estimated as the metric of hysteresis. Considering this metric and each stimulation direction separately, the perceptual transitions of each participant between each emotion pair (average of 10-second dynamic transition trials) were classified as showing positive, negative, or no hysteresis. When no difference existed between the dynamic perceptual switch point and the one occurring in case of no perceptual history (dynamic inflection = static inflection), we assumed that no perceptual hysteresis occurred. When this difference was greater than 0, revealing a latter perceptual switch during the dynamic transitions than during the static control (dynamic inflection > static inflection), we assumed positive hysteresis occurred. Whereas when this difference was less than 0, revealing an earlier perceptual switch during the dynamic transitions than during the static control, we assumed negative hysteresis occurred (dynamic inflection < static inflection).

### Imaging data processing

Data processing was performed using BrainVoyager v21.4 (Brain Innovation, The Netherlands) and MATLAB (R2019a, The MathWorks) custom-made scripts. Pre-processing included slice-scan time correction, 3D head-motion correction, and temporal high-pass filtering (GLM-Fourier, 3 cycles). Data were normalized into MNI-152 space (Fonov et al., 2011). For the first-level analysis, activation maps were created using a General Linear Model (GLM), with predictors for each experimental condition and confound predictors from six detrended head motion parameters (3 translation, 3 rotation). Second-level group analyses were performed using Random Effects (RFX) analysis to allow for population inferences.

### Imaging data analysis

#### Static control runs

The static control runs, which required emotion discrimination via the classification of the perceived emotion in each static image, were used as task-related functional localizer. An FFX-GLM analysis was performed considering all static control runs per subject and contrasting facial images *versus* the fixation crosses intervals. The resulting activity map, with a threshold at the statistical value of *q*(FDR) = 0.05, allowed us to define regions-of-interest (ROIs) bilaterally in the anterior insula – our main ROI, and the fusiform face area (FFA) and the superior temporal sulcus (STS), in which the representational space for facial identity and expressions are stored, respectively (Bernstein et al., 2018; Duchaine & Yovel, 2015; Said et al., 2011).

#### Dynamic transitions runs

A) Activity Analysis

To investigate the neural network underlying perceptual hysteresis, we first performed RFX-GLM analyses within our ROIs considering all dynamic runs. Taking into account our previous study (Verdade et al., 2020) and the dominance of positive hysteresis in the perception of dynamic emotion expressions, we focused our analyses on this positive signature of perceptual hysteresis. To identify which brain regions are modulated by the predominance of positive hysteresis, we contrasted the stimulation direction which showed predominant positive hysteresis *versus* the one showing less dominance of positive hysteresis. This means, we considered pairs of emotions where, for example, there was a stimulation direction in which all participants except one showed positive hysteresis, whereas the in the other we found similar number of participants showing positive and negative hysteresis.

Then, to further investigate the neural correlates of positive hysteresis, we estimated the percentage of change of the BOLD signal over time for the ROIs which revealed to be modulated by positive hysteresis. This analysis was performed for each direction of stimulation and each emotion pair during trials where positive hysteresis dominated and compared to trials where there was no predominance of either signature of hysteresis. Time courses of each ROI were extracted for each dynamic transitions run and percent changes of the signal were calculated with respect to the voxels time course mean value and averaged within and across participants.

B) Connectivity Analysis

To assess functional connectivity during perceptual hysteresis, we performed psychophysiological interaction (PPI) analysis, which identifies a task-specific increase in the exchange of information between brain regions. We computed the generalized PPI (gPPI) approach proposed by McLaren et al. (2012), which better performs for studies with multiple conditions than the initially PPI analysis proposed by Friston et al. (1997). Following our initial hypothesis of the right anterior insula as a critical region in the network to which perceptual hysteresis may map into in the context of emotion recognition, this region was used as seed ROI. We employed the BrainVoyager PPI plugin per participant taking into account the motion parameters as confounds. The gPPI approach implemented in BrainVoyager’s PPI plugin models the interaction between the psychological context and the brain activity at the haemodynamic response function level instead of the neural activity level. Therefore, it does not apply deconvolution, as suggested by Gitelman and colleagues (2003), which would be especially important for event-related analysis, rather than block design analysis, as the one followed in our study. In short, we extracted the mean activity of the seed ROI for each TR, which was *z*-transformed before to be multiplied TR by TR with the task time course based on the dynamic runs protocol. Before being multiplied by the ROI time course, the task time course was convolved with the haemodynamic response function. The plugin then computed all of the predictors and confounds and save them into a GLM matrix. Given the typically low statistical power of the PPI analysis, we decided to analyse data by contrasting directions showing predominant positive hysteresis *versus* directions where positive hysteresis was not predominant, regardless of the emotion pair. This allowed us to identify regions whose connectivity with the right anterior insula was dependent on the prevalence of positive hysteresis.

Finally, we examined perceptual hysteresis correlation dynamics over time during our dynamic trials, taking into account a hemodynamic delay of six seconds. We considered the directions where positive hysteresis dominated and the directions where there was no predominance of either signature of hysteresis. A centred sliding window of ten seconds was used to estimate the time courses of partial Spearman’s correlation between the right anterior insula and the medial prefrontal cortex (mPFC), which has been suggested to underlie perceptual hysteresis, conditioned on the time courses of a ROI with noisy signal (unrelated spherical ROI with 257 voxels defined on the white matter). All correlation coefficients were converted to *z*-scores using Fisher’s z-transformation.

### Statistical analysis

Data were tested for normality using a Kolmogorov–Smirnov test with an *α*-level of 0.05 before running further statistical analyses. To analyse the significance of the hysteresis metrics, the perceptual switch difference between each direction and the control case was compared using a non-parametric Wilcoxon test. Differences in the percentage of signal change between the two directions were also calculated using a non-parametric Wilcoxon test. Correlation dynamics at the group level differences were evaluated using a paired sample Wilcoxon signed-rank test (2-tailed). All statistical analyses were performed with SPSS Statistics V22.0 (IBM, Armonk, NY) and MATLAB (R2019a, The MathWorks).

## Results

### Perceptual hysteresis on emotion recognition: behavioral results

**Fig. 3:**
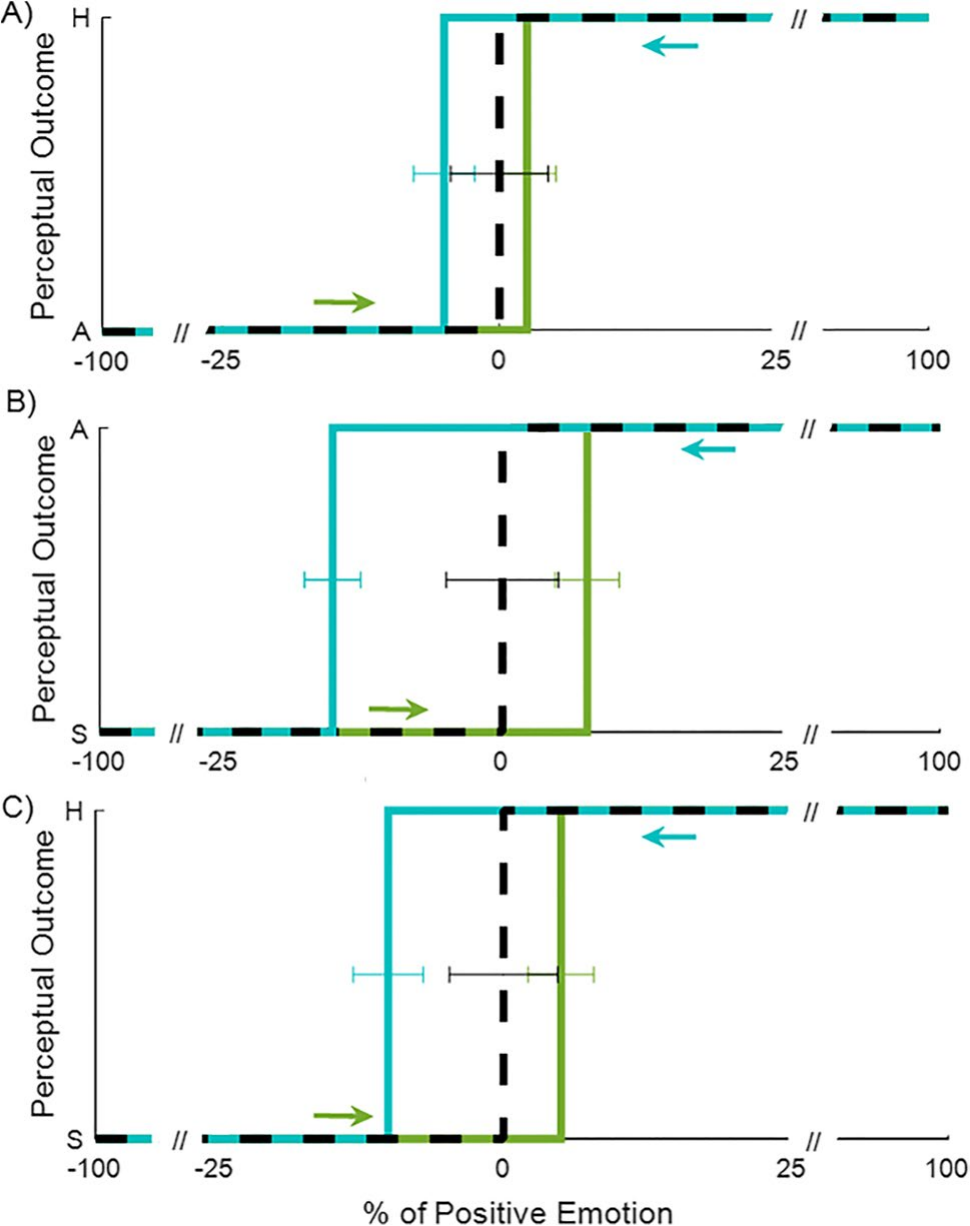
**Perceptual hysteresis on emotion recognition during dynamic transitions**. Group results of the average perceptual inflection point (mean of transition points at each neutral interval border) for both directions 1 (green) and 2 (blue) and the respective static control condition (discontinuous black curve). ***a*** Anger-happiness pair; ***b*** sadness-anger pair; ***c*** sadness-happiness pair. *A* = anger; *H* = happiness; *S* = sadness. Results are shown in terms of percentage of positive emotion (anger considered as such for visualization purposes), which was used to parametrize each dynamic transition and varied in a control manner. Error bars correspond to within-subjects SEM

We found perceptual hysteresis for all three pairs of emotions tested, as the mean transition point depended on the trajectory. The timing of perceptual switches between both directions of stimulation (Fig. 3) were significantly different for all pairs of emotions (pair anger-happiness: *Z* = −3.181, *p =* 0.001; pair sadness-anger: *Z* = −3.621, *p =* 0.0003; pair sadness-happiness: *Z* = −3.243, *p =* 0.001). The greatest difference was found for the pair sadness-anger, replicating our previous behavioral study (Verdade et al., 2020).

Perceptual hysteresis was then classified based on the difference of the perceptual switch to the opposing percept in each direction and the switch of the static control curve (no history). Wilcoxon signed-rank tests revealed significant differences for these hysteresis values between the two stimulation directions for the pairs sadness-anger (Z = −2.563, *p* = 0.010) and sadness-happiness (Z = −2.443, *p* = 0.015) being the positive hysteresis stronger in stimulation direction 2 than in stimulation direction 1. However, this was not the case for the pair anger-happiness (Z = −0.742, *p* = 0.458) where the predominance of positive hysteresis was similar in both stimulation directions.

In transitions between neutral expressions, used as control for the dynamic transition per se, as expected, the number of trials in which two responses were given (indicating a transition to and from neutral) was less than 5% (4.72 ± 2.53%), thus not contributing to the recorded patterns of hysteresis.

### Neurophysiological results

#### Regions-of-interest localization

**Fig. 4:**
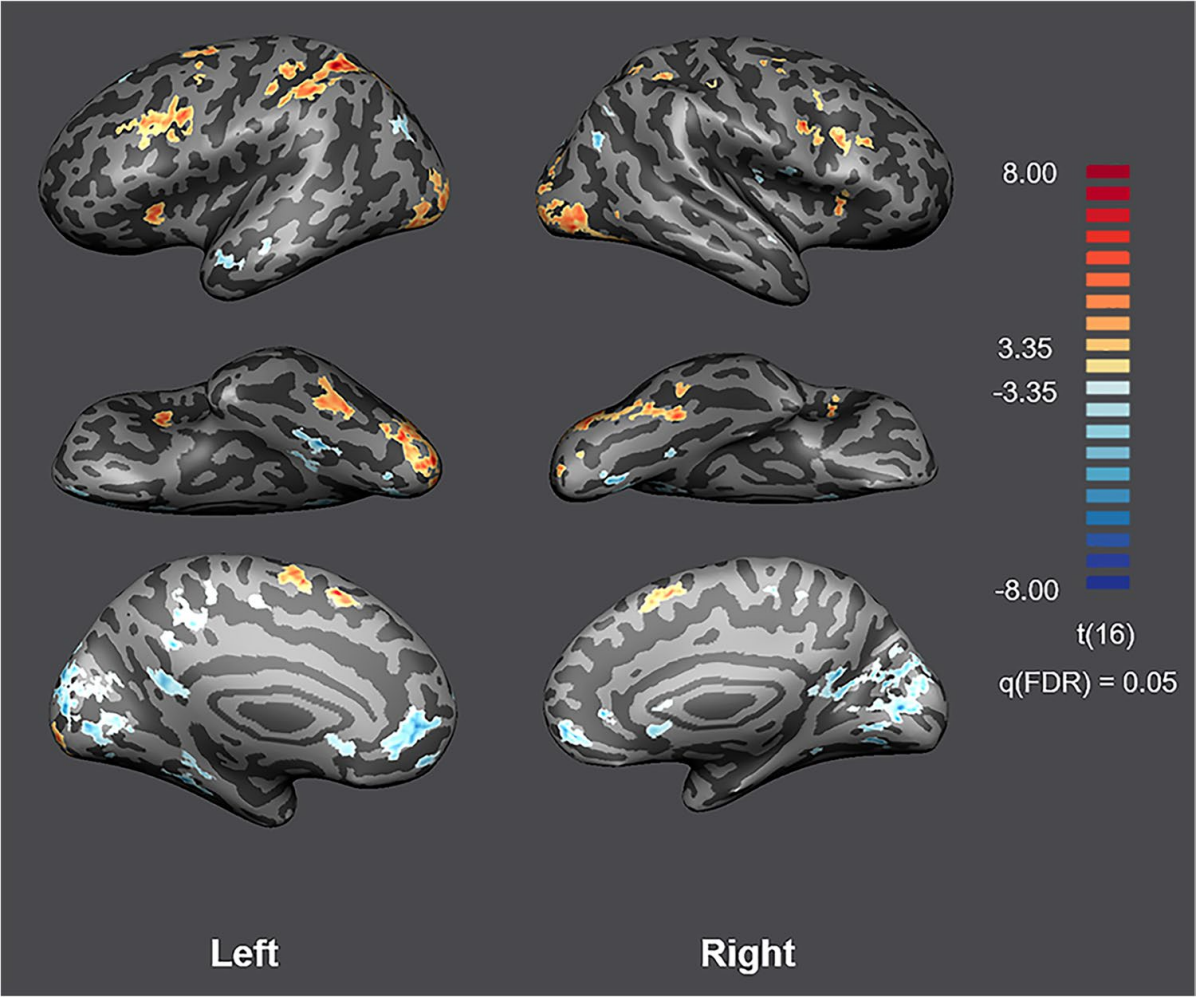
**RFX-GLM activation map of the static control runs when contrasting facial images and the fixation cross.** The contrast reveals task-related regions of interest at the group level, such as the right and left anterior insula, FFA, and STS. Group statistical map is presented at *q*(FDR) = 0.05

Our localizer approach allowed us to identify at the right and left hemispheres the anterior insula, FFA, and STS. These ROIs were defined considering the FFX-GLM group activity map of the static control runs by contrasting all stimulation conditions (faces) *versus* rest periods (fixation cross) per participant. The MNI coordinates of the selected regions and the number of voxels are summarized in Supplementary Table 1. For illustrative purposes, Fig. 4 displays the group activity map resulting the RFX-GLM analysis at *q*(FDR = 0.05).

#### Neural signatures of positive hysteresis

In light of the Wilcoxon signed-rank tests on hysteresis signatures, we first performed our analyses on the pairs with asymmetric dominance of hysteresis (sadness-anger and sadness-happiness), which allowed us to study the effect of the different signatures of perceptual hysteresis on the absolute activation of our ROIs (considering the direction showing predominant positive hysteresis and the direction showing no prevalent signature of hysteresis). In the sadness-anger pair, the ROI RFX-GLM analysis revealed that the right FFA (*t* = 2.48; *p* = 0.024, FDR-corrected) and the right STS (*t* = 2.78; *p* = 0.013, FDR-corrected) responses were significantly higher when positive hysteresis was prevalent than when there was no predominance. Moreover, in the pair sadness-happiness, we found a smaller right anterior insula response when positive hysteresis was predominant (*t* = −2.17; *p* = 0.044, FDR-corrected).

**Fig. 5:**
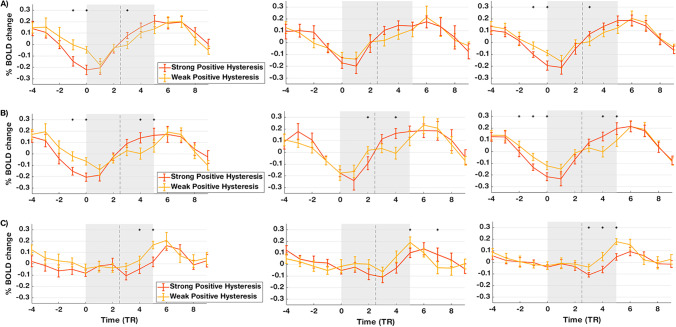
**BOLD percent of signal change during dynamic transitions trials.** Time course of brain responses during predominant positive hysteresis (orange) and in cases where this signature did not dominate (yellow) were averaged within and across all participants, for the right FFA (***a***), the right STS (***b***), and the right anterior insula (***c***). Differences between percent signal changes in the two types of samples are shown for the sadness-anger (left panel) and sadness-happiness (middle panel) pairs, and also irrespective of the pair of emotions (right panel). Interval highlighted in grey corresponds to the dynamic perceptual transitions and the discontinuous black curve corresponds to the moment of 100% neutral. Stars in the graph denote significant differences in BOLD percent of signal change between the two types of trials (*p* < 0.05). Strong positive hysteresis differentially modulates the face processing visual regions (right FFA and ring STS) and the right anterior insula. Error bars correspond to within-subjects SEM

To investigate the neural correlates of positive hysteresis over time, we calculated the BOLD percent signal change during trials of the directions showing predominant positive hysteresis and trials of the directions showing less dominance. Considering the ROI RFX-GLM results and the right hemisphere in all our three regions of interest, we focused our analyses on the right FFA, the right STS, and the right anterior insula (Fig. 5). Overall, significant differences (*p* < 0.05) in the BOLD signal changes between the two directions were found in all three regions in both pairs (Fig. 5, left and middle panel), and also when ignoring the emotion pair tested (Fig. 5, right panel). Before the perceptual transition, all regions responded similarly, while during the perceptual transition, the right FFA and right STS revealed an opposite response pattern to the right anterior insula. Both right FFA and STS responded more to strong positive hysteresis than weak positive hysteresis. The right anterior insula presented a higher response for weak positive hysteresis than strong positive hysteresis. These patterns were found for both sadness-anger and sadness-happiness pairs but were most evident for the former.

#### Neural network of perceptual hysteresis

**Fig. 6:**
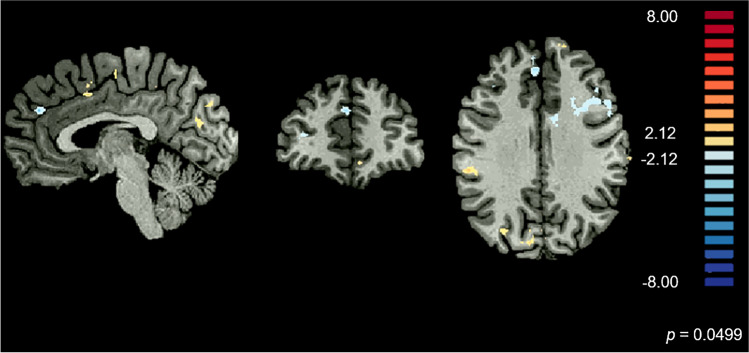
**Generalized psychophysiological interaction analysis (gPPI) of the neural correlates of dynamic transitions between pairs of emotions when positive hysteresis was dominant.** Functional connectivity between the right anterior insula, which was used as seed ROI, and the mPFC (*x*, *y*, *z* MNI coordinates: 4, 45, 29) was decresead when positive hysteresis was dominant (*p* = 0.049)

Considering our hypothesis that the right anterior insula plays a critical role in the mechanisms of perceptual hysteresis, we conducted a hypothesis-driven PPI analysis to further investigate its connectivity with additional brain regions that may contribute to the neural network of perceptual hysteresis. When contrasting stimulation directions based on the predominance of positive hysteresis, regardless of the emotion pair, we found that the right anterior insula showed lower connectivity with mPFC (*x*, *y*, *z* MNI coordinates: 4, 45, 29) when positive hysteresis dominated (*p* < 0.05) (Fig. 6).

**Fig. 7:**
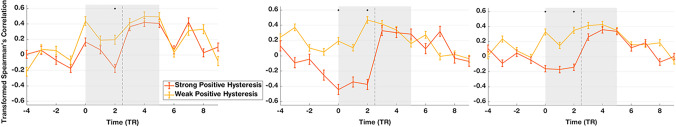
**Group analysis of the time varying correlation between the right anterior insula and the mPFC during dynamic transitions between pairs of emotions.** Greater correlations were observed for directions where positive hysteresis did not dominate (yellow), for both the pairs sadness-anger (left panel) and sadness-happiness (middle panel), and also irrespective of the pair of emotions tested (right panel). Interval highlighted in grey corresponds to the dynamic perceptual transitions and the discontinuous black curve corresponds to the moment of 100% neutral. Stars in the graph denote significant differences in partial Spearman’s correlations between the two types of trials (*p* < 0.05). Error bars correspond to within-subjects SEM

**Fig. 8:**
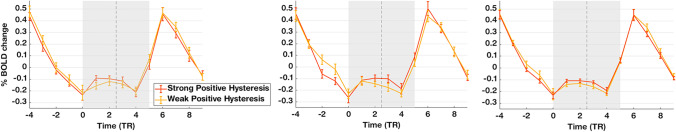
**BOLD percent signal change in the mPFC during dynamic transitions trials.** Percent of signal changes during trials of directions where positive hysteresis was predominant (orange) and trials of directions where neither positive or negative hysteresis dominated (yellow) for the pair sadness-anger (left panel), the pair sadness-happiness (middle panel), and ignoring the emotion pair tested (right panel). No significant differences were registered between the two types of trials in either the pair of emotions. Interval highlighted in grey corresponds to the dynamic perceptual transitions and the discontinuous black curve corresponds to the moment of 100% neutral. Error bars correspond to within-subjects SEM

We further investigated how the dynamics of the temporal correlation between these two regions was influenced by the predominance of positive hysteresis. A sliding window was used to track an event-related time course of the correlation between the right anterior insula and the mPFC along the stimulus visualization for trials of directions where positive hysteresis was dominant and trials of directions where there was no dominance of either signature. Figure 7 shows these results for both sadness-anger and sadness-happiness pairs, and the same analysis when ignoring the pair of emotion. In both pairs we found significantly greater correlations between the two regions when positive hysteresis was not predominant, and this was also the case when ignoring the pair of emotion being tested. We ruled out that this was due to different activation patterns on the mPFC depending on the predominance of positive hysteresis, because the Wilcoxon signed-rank test did not show statistically significant differences between the two types of cases in either pair or when ignoring the pair tested (Fig. 8).

Overall, the right anterior insula showed a pattern of activity depending on the predominance of the positive signature of perceptual hysteresis. Moreover, although the activity of the mPFC did not show this dependency, functional connectivity between these two regions was modulated by it. The temporal correlation of the right anterior insula and the mPFC revealed to be lower when positive hysteresis dominated perception.

## Discussion

In the current study, we investigated the neural mechanisms underlying perceptual hysteresis in the context of facial emotion recognition, and in particular the role of early visual high-level regions and the insular cortex in perceptual persistence and decision. To do so, we used a visual paradigm consisting of reality-based transitions from a *source* to a *target* emotional expression, always passing through a neutral expression.

The participants’ perception of the dynamic emotional transitions here tested showed to be dependent on the direction of stimulation thus revealing perceptual hysteresis. Moreover, we found dominant positive hysteresis as participants tended to persist longer on the original percept, when compared with the no history situation, as earlier seen in our previous behavioural study (Verdade et al., 2020).

Our neuroimaging data revealed right FFA, right STS, and right anterior insula modulation by positive hysteresis predominance. This is in agreement with the described FFA and STS involvement in facial emotion processing in the human brain (De Winter et al., 2015; Fan et al., 2020; Kanwisher et al., 1997; Karolis et al., 2019; Nakamura et al., 2014; Wang et al., 2020). Our results further provide evidence for the anterior insular cortex modulation during perceptual hysteresis, adding to the debate on its role in perception-driven processing (Craig, 2009; Eckert et al., 2009; Gu et al., 2013; Wicker et al., 2003). The anterior insula has been shown to play a critical role in high-level cognitive control and attentional processes (Chang et al., 2013; Menon & Uddin, 2010; Touroutoglou et al., 2012).

As one of the core nodes of the salience network, the anterior insula has been described to work in the integration of sensory information and to play a causal role in mediating cognitive processing (Menon, 2015; Menon & Uddin, 2010; Seeley, 2019; Uddin et al., 2017), thus being crucial in perceptual decision-making. Here, we tested the hypothesis of the right anterior insula being a core hub in perceptual hysteresis. Our results showed the response of the right anterior insula to be dependent on the dominance of positive hysteresis, which confirms our hypothesis and is in accordance with previous studies showing an involvement of the right anterior insula in hysteresis in visual perception (Sayal et al., 2020; Schwiedrzik et al., 2014). We now show its involvement in hysteresis of facial emotion recognition, reinforcing that the right anterior insula plays a crucial role in this phenomenon in overall perception.

We further estimated connectivity of the right anterior insula with the remaining brain regions in perceptual hysteresis by performing functional connectivity analyses. Our results showed lower interaction of the right anterior insula with the mPFC when positive hysteresis was predominant. Importantly, this lower connectivity was not due to a differential activation of the mPFC, because activity in this region did not show to be dependent on the prevalence of positive hysteresis, as no significant differences between the two types of cases (strong and weak positive hysteresis) were found. The mPFC, functionally divided into ventral and dorsal regions, plays a critical role in both memory and decision making, guiding adaptive behaviour (Euston et al., 2012). A great body of neurophysiological evidence shows that the mPFC acts as an action-outcome predictor, integrating salient inputs and mapping them onto the appropriate choices based on past experience (Alexander & Brown, 2011). The mPFC is strongly connected with the anterior insula via the salience network, and although the exact role of this connectivity pattern remains elusive, functional MRI studies have shown a possible dependence of the mPFC role as an choice-outcome predictor on the inputs provided by the anterior insula (Billeke et al., 2020; Ebisch et al., 2013). Moreover, this region has earlier been reported to behave as a central node of high-order regions related to perceptual stabilization in the context of hysteresis (Schwiedrzik et al., 2014). In particular, it was recently shown that this region is functionally specific for perceptual memory, integrating current sensory information with prior percepts and stabilizing visual experience against the perceptual variability (Schwiedrzik et al., 2018).

It is known that dmPFC activity in the high gamma frequency band (70–150 Hz) correlates with perceptual memory (Castelhano et al., 2017). This effect has been found to be anatomically specific to dmPFC and functionally specific for memories of preceding percepts. Furthermore, dmPFC appears to play a causal role, as patients with lesions in this area have shown impaired perceptual memory. Thus, dmPFC is believed to integrate current sensory information with prior percepts, stabilizing visual experience against the perpetual variability of our surroundings.

Our results shed light on the anterior insula role as an integral hub of sensory information and are consistent with a possible causal relationship of the mPFC role as an choice-outcome predictor on the inputs provided by the anterior insula in the perceptual hysteresis phenomenon (Billeke et al., 2020; Ebisch et al., 2013). The significantly higher connectivity to the mPFC when positive hysteresis was not prevalent may reflect a greater effort in integrating weak or ambiguous sensory information, which in turn results in a higher recruitment of the mPFC, and a reinforcement of the interconnectivity of the anterior insula to this region to resolve perceptual ambiguity. Perception has been modelled in terms of Bayesian probability estimation (Fletcher & Frith, 2009; Kersten & Yuille, 2003) as an interplay between bottom-up (sensory) information and top-down (attention and memory) mechanisms. Two mechanisms have been reported to underlie perceptual hysteresis: one responsible for maintaining the current percept even when stimulus parameters favour an alternative one, and the other forcing the percept to switch early on (Fritsche et al., 2017; Kleinschmidt et al., 2002; Liaci et al., 2018; Sayal et al., 2020; Schwiedrzik et al., 2014). While earlier research describes these two mechanisms as arising from the same neural process (Blake et al., 2003; Chen & He, 2004; Gepshtein & Kubovy, 2005), recent neurophysiological studies have provided evidence that not only do they map into hierarchically distinct networks (Schwiedrzik et al., 2014) but that the continuous competition between them ultimately determines the perceptual hysteresis effect (Kleinschmidt et al., 2002; Sayal et al., 2020).

We were able to confirm the involvement of the anterior insula in hysteresis using a facial emotion recognition paradigm, which we believe reinforces its involvement in the neural circuitry to which hysteresis maps into in overall perception. Our results are consistent with the hypothesis of higher-order regions being involved in perceptual stabilization and decision in the context of perceptual hysteresis (Kleinschmidt et al., 2002; Sayal et al., 2020; Schwiedrzik et al., 2014). Moreover, our functional connectivity analyses revealed an interplay between the right anterior insula and the mPFC, emphasizing the view of the anterior insula working as an integral hub between the integration of sensory information and higher-order cognitive networks, in the decision-making process. Finally, the differential connectivity between these two regions depending on the prevalence of positive hysteresis now adds evidence to the discussion of the hypothesis of differential network recruitment for the two mechanisms underlying perceptual hysteresis.

Nonetheless, it is important to acknowledge an important limitation of the present study, namely the fact that our paradigm did not allow to investigate dominance of negative hysteresis given the perceptual nature of facial emotion recognition (Verdade et al., 2020). Thus, future studies should unveil the specific involvement of the anterior insula in perceptual hysteresis, especially in paradigms that allow to investigate its role in cases where the two forms dominance occur. Also, given the debate of two distinct mechanisms underlying perceptual hysteresis effects, functional connectivity analysis could further reveal the nature of the relationship of the anterior insula and mPFC in this context and shed light to the underlying networks of these mechanisms. Moreover, as these two regions are also part of the central autonomous network (Beissner et al., 2013), it would be relevant to evaluate the contribution of arousal to their connectivity modulation by perceptual hysteresis.

Taking into account the present results, we add to the evidence that hysteresis paradigms allow to assess the dynamics of perception and short term visual memory (Fritsche et al., 2017; Liaci et al., 2018; Liberman et al., 2018; Mei et al., 2019; Sacharin et al., 2012; Witthoft et al., 2018). Thus, understanding the phenomenon of perceptual hysteresis and its underlying neural circuitry may serve as body of research to investigate perceptual inflexibility in many neuropsychiatric disorders, such as autism and schizophrenia (Barbalat et al., 2012; Behrmann et al., 2006; Hadad & Schwartz, 2019; Martin et al., 2014), ultimately providing insights into the compromised cognitive processes of these disorders.

## Conclusions

Our results show that changing facial emotion expressions leads to dominant positive perceptual hysteresis and that early visual regions are related to perceptual persistence. We found neural evidence for the involvement of the right anterior insula in perceptual hysteresis. We also found that functional connectivity between this region and the mPFC was significantly lower when positive hysteresis was predominant.

We believe that our findings confirm the involvement of high-order regions in the process of perceptual hysteresis, particularly the anterior insula, and face processing visual regions, such as FFA and STS. Moreover, our results support the hypothesis of the former being an integral hub of sensory information for hierarchically higher regions involved in decision-making during perceptual hysteresis. Further neurophysiological research is needed to better understand the relative role of lower and higher-order regions in visual perception hysteresis.

## Supplementary information

ESM 1 (DOCX 18.7 kb)

## Footnotes

1 An example of a dynamic transition from the pair of emotions anger-happiness, used in the task, is available in the following link: https://bit.ly/2DmkdKc.

## References

[CR1] Alexander WH, Brown JW (2011). Medial prefrontal cortex as an action-outcome predictor. Nature Neuroscience.

[CR2] Barbalat G, Rouault M, Bazargani N, Shergill S, Blakemore SJ (2012). The influence of prior expectations on facial expression discrimination in schizophrenia. Psychological Medicine.

[CR3] Behrmann M, Thomas C, Humphreys K (2006). Seeing it differently: Visual processing in autism. Trends in Cognitive Sciences.

[CR4] Beissner F, Meissner K, Bär KJ, Napadow V (2013). The autonomic brain: An activation likelihood estimation meta-analysis for central processing of autonomic function. The Journal of Neuroscience.

[CR5] Bernstein M, Erez Y, Blank I, Yovel G (2018). An Integrated Neural Framework for Dynamic and Static Face Processing. Scientific Reports.

[CR6] Billeke P, Ossandon T, Perrone-Bertolotti M, Kahane P, Bastin J, Jerbi K, Lachaux JP, Fuentealba P (2020). Human Anterior Insula Encodes Performance Feedback and Relays Prediction Error to the Medial Prefrontal Cortex. Cerebral Cortex.

[CR7] Blake R, Sobel KV, Gilroy LA (2003). Visual motion retards alternations between conflicting perceptual interpretations. Neuron.

[CR8] Castelhano, J., Duarte, I. C., Wibral, M., Rodriguez, E., & Castelo-Branco, M. (2014). The dual facet of gamma oscillations: separate visual and decision making circuits as revealed by simultaneous EEG/fMRI. *Human Brain Mapping,**35*(10), 5219–35. 10.1002/hbm.22545 10.1002/hbm.22545 PMC6869291 24839083

[CR9] Castelhano J, Duarte IC, Abuhaiba SI, Rito M, Sales F, Castelo-Branco M (2017). Cortical functional topography of high-frequency gamma activity relates to perceptual decision: An intracranial study. PLoS One.

[CR10] Chand GB, Dhamala M (2016). The salience network dynamics in perceptual decision-making. Neuroimage.

[CR11] Chang LJ, Yarkoni T, Khaw MW, Sanfey AG (2013). Decoding the role of the insula in human cognition: Functional parcellation and large-scale reverse inference. Cerebral Cortex.

[CR12] Chen X, He S (2004). Local factors determine the stabilization of monocular ambiguous and binocular rivalry stimuli. Current Biology.

[CR13] Craig AD (2009). How do you feel - now? The anterior insula and human awareness. Nature Reviews. Neuroscience.

[CR14] De Winter FL, Zhu Q, Van den Stock J, Nelissen K, Peeters R, de Gelder B, Vanduffel W, Vandenbulcke M (2015). Lateralization for dynamic facial expressions in human superior temporal sulcus. Neuroimage.

[CR15] Direito B, Lima J, Simões M, Sayal A, Sousa T, Lührs M, Ferreira C, Castelo-Branco M (2019). Targeting dynamic facial processing mechanisms in superior temporal sulcus using a novel fMRI neurofeedback target. Neuroscience.

[CR16] Dubé SP (1997). Visual bases for the perception of facial expressions: A look at some dynamic aspects.

[CR17] Duchaine B, Yovel G (2015). A revised neural framework for face processing. Annual Review of Vision Science.

[CR18] Ebisch SJH, Mantini D, Romanelli R, Tommasi M, Perrucci MG, Romani GL, Colom R, Saggino A (2013). Long-range functional interactions of anterior insula and medial frontal cortex are differently modulated by visuospatial and inductive reasoning tasks. Neuroimage.

[CR19] Eckert MA, Menon V, Walczak A, Ahlstrom J, Denslow S, Horwitz A, Dubno JR (2009). At the heart of the ventral attention system: The right anterior insula. Human Brain Mapping.

[CR20] Ekman P, Friesen WV (1978). Facial Action Coding System: A Technique for the Measurement of Facial Movement.

[CR21] Euston DR, Gruber AJ, McNaughton BL (2012). The Role of Medial Prefrontal Cortex in Memory and Decision Making. Neuron.

[CR22] Fan X, Wang F, Shao H, Zhang P, He S (2020). The bottom-up and top-down processing of faces in the human occipitotemporal cortex. Elife.

[CR23] Fletcher PC, Frith CD (2009). Perceiving is believing: A Bayesian approach to explaining the positive symptoms of schizophrenia. Nature Reviews. Neuroscience.

[CR24] Fonov V, Evans AC, Botteron K, Almli CR, McKinstry RC, Collins DL (2011). Unbiased average age-appropriate atlases for pediatric studies. Neuroimage.

[CR25] Friston KJ, Buechel C, Fink GR, Morris J, Rolls E, Dolan RJ (1997). Psychophysiological and modulatory interactions in neuroimaging. Neuroimage.

[CR26] Fritsche, M., Mostert, P., & de Lange, F. P. (2017). Opposite effects of recent history on perception and decision. *Current Biology*. 10.1016/j.cub.2017.01.006 10.1016/j.cub.2017.01.006 28162897

[CR27] Gazzaley, A., Rissman, J., & D’Esposito, M. (2004). Functional connectivity during working memory maintenance. *Cognitive, Affective, & Behavioral Neuroscience, 4*(4), 580–599. 10.3758/cabn.4.4.580 10.3758/cabn.4.4.580 15849899

[CR28] Gepshtein S, Kubovy M (2005). Stability and change in perception: Spatial organization in temporal context. Experimental Brain Research.

[CR29] Gu X, Hof PR, Friston KJ, Fan J (2013). Anterior insular cortex and emotional awareness. The Journal of Comparative Neurology.

[CR30] Hadad BS, Schwartz S (2019). Perception in autism does not adhere to weber’s law. Elife.

[CR31] Hock, H. S., & Schöner, G. (2011). Measuring perceptual hysteresis with the modified method of limits: Dynamics at the threshold. In Solomon, J. A. (Ed.), *Fechner’s legacy in psychology: 150 years of elementary psychophysics* (pp. 63–85). BRILL. 10.1163/187847510X503597 20550825

[CR32] Hsu SM, Wu ZR (2019). The roles of preceding stimuli and preceding responses on assimilative and contrastive sequential effects during facial expression perception. Cognition & Emotion.

[CR33] Jiles, D. C., & Atherton, D. L. (1986). Theory of ferromagnetic hysteresis. *Journal of Magnetism and Magnetic Materials*. 10.1016/0304-8853(86)90066-1

[CR34] Kamachi M, Bruce V, Mukaida S, Gyoba J, Yoshikawa S, Akamatsu S (2013). Dynamic properties influence the perception of facial expressions. Perception.

[CR35] Kanwisher N, McDermott J, Chun MM (1997). The fusiform face area: A module in human extrastriate cortex specialized for face perception. The Journal of Neuroscience.

[CR36] Karolis, V. R., Corbetta, M., & Thiebaut de Schotten, M. (2019). The architecture of functional lateralisation and its relationship to callosal connectivity in the human brain. *Nature Communications, 10*(1417). 10.1038/s41467-019-09344-1 10.1038/s41467-019-09344-1 PMC6441088 30926845

[CR37] Kersten D, Yuille A (2003). Bayesian models of object perception. Current Opinion in Neurobiology.

[CR38] Kleinschmidt A, Büchel C, Hutton C, Friston KJ, Frackowiak RSJ (2002). The neural structures expressing perceptual hysteresis in visual letter recognition. Neuron.

[CR39] Kobayashi H, Hara F. (1993). Dynamic recognition of basic facial expressions by discrete-time recurrent neural network. In: Proceedings of 1993 International Conference on Neural Networks (IJCNN-93-Nagoya, Japan). Nagoya, Japan. p. 155–158.

[CR40] LaBar KS, Crupain MJ, Voyvodic JT, McCarthy G (2003). Dynamic perception of facial affect and identity in the human brain. Cerebral Cortex.

[CR41] Lamichhane, B., Adhikari, B. M., & Dhamala, M. (2016). The activity in the anterior insulae is modulated by perceptual decision-making difficulty. *Neuroscience*. 10.1016/j.neuroscience.2016.04.016 10.1016/j.neuroscience.2016.04.016 27095712

[CR42] Liaci E, Fischer A, Atmanspacher H, Heinrichs M, Van Elst LT, Kornmeier J (2018). Positive and negative hysteresis effects for the perception of geometric and emotional ambiguities. PLoS One.

[CR43] Liberman A, Manassi M, Whitney D (2018). Serial dependence promotes the stability of perceived emotional expression depending on face similarity. Attention, Perception, & Psychophysics.

[CR44] Lopresti-Goodman, S. M., Turvey, M. T., & Frank, T. D. (2013). Negative hysteresis in the behavioral dynamics of the affordance “graspable”. *Attention, Perception, & Psychophysics*. 10.3758/s13414-013-0437-x 10.3758/s13414-013-0437-x 23471744

[CR45] Lucey P, Cohn JF, Kanade T, Saragih J, Ambadar Z, Matthews I. (2010). The extended Cohn-Kanade dataset (CK+): A complete dataset for action unit and emotion-specified expression. In: 2010 IEEE Computer Society Conference on Computer Vision and Pattern Recognition - Workshops, CVPRW 2010. San Francisco, CA. p. 94–101.

[CR46] Luenberger DG (1979). Introduction to Dynamic Systems Theory, Models, and Applications.

[CR47] Martin JR, Dezecache G, Pressnitzer D, Nuss P, Dokic JÔ, Bruno N, Pacherie E, Franck N (2014). Perceptual hysteresis as a marker of perceptual inflexibility in schizophrenia. Consciousness and Cognition.

[CR48] McLaren DG, Ries ML, Xu G, Johnson SC (2012). A generalized form of context-dependent psychophysiological interactions (gPPI): A comparison to standard approaches. Neuroimage.

[CR49] Mei G, Chen S, Dong B (2019). Working memory maintenance modulates serial dependence effects of perceived emotional expression. Frontiers in Psychology.

[CR50] Menon V (2015). Salience Network. *Brain Mapping: An Encyclopedic Reference*.

[CR51] Menon V, Uddin L (2010). Saliency, switching, attention and control: A network model of insula function. Brain Structure & Function.

[CR52] Nakamura A, Maess B, Knösche TR, Friederici AD (2014). Different hemispheric roles in recognition of happy expressions. PLoS One.

[CR53] Orbach J, Ehrlich D, Heath HA (1963). Reversibility of the Necker Cube. I. An examination of the concept of “satiation of orientation”. Perceptual and Motor Skills.

[CR54] Pearson J, Brascamp J (2008). Sensory memory for ambiguous vision. Trends in Cognitive Sciences.

[CR55] Pessoa L, Padmala S (2005). Quantitative prediction of perceptual decisions during near-threshold fear detection. Proceedings of the National Academy of Sciences of the United States of America.

[CR56] Pisarchik AN, Jaimes-Reátegui R, Magallón-García CDA, Castillo-Morales CO (2014). Critical slowing down and noise-induced intermittency in bistable perception: Bifurcation analysis. Biological Cybernetics.

[CR57] Russell JA (1980). A circumplex model of affect. Journal of Personality and Social Psychology.

[CR58] Sacharin, V., Sander, D., & Scherer, K. R. (2012). The perception of changing emotion expressions. *Cognition & Emotion*. 10.1080/02699931.2012.656583 10.1080/02699931.2012.656583 22550942

[CR59] Said CP, Haxby JV, Todorov A (2011). Brain systems for assessing the affective value of faces. Philosophical Transactions of the Royal Society B: Biological Sciences.

[CR60] Sato W, Kochiyama T, Yoshikawa S, Naito E, Matsumura M (2004). Enhanced neural activity in response to dynamic facial expressions of emotion: An fMRI study. Cognitive Brain Research.

[CR61] Sato W, Kochiyama T, Uono S, Yoshikawa S (2010). Amygdala integrates emotional expression and gaze direction in response to dynamic facial expressions. Neuroimage.

[CR62] Sayal, A., Sousa, T., Duarte, J. V., Costa, G. N., Martins, R., & Castelo-Branco, M. (2020). Identification of competing neural mechanisms underlying positive and negative perceptual hysteresis in the human visual system. *Neuroimage, 221*(July). 10.1016/j.neuroimage.2020.117153 10.1016/j.neuroimage.2020.117153 32659351

[CR63] Schwiedrzik CM, Ruff CC, Lazar A, Leitner FC, Singer W, Melloni L (2014). Untangling perceptual memory: Hysteresis and adaptation map into separate cortical networks. Cerebral Cortex.

[CR64] Schwiedrzik, C. M., Sudmann, S. S., Thesen, T., Wang, X., Groppe, D., Mégevand, P., Doyle, W., Mehta, A. D., Devinsky, O., & Melloni L. (2018). Medial prefrontal cortex supports perceptual memory. *Current Biology, 28*(18), PR1094-PR1095. 10.1016/j.cub.2018.07.066 30253147

[CR65] Seeley WW (2019). The salience network: A neural system for perceiving and responding to homeostatic demands. The Journal of Neuroscience.

[CR66] Singer T, Critchley HD, Preuschoff K (2009). A common role of insula in feelings, empathy and uncertainty. Trends in Cognitive Sciences.

[CR67] Stoner EC, Wohlfarth EP (1991). A mechanism of magnetic hysteresis in heterogeneous alloys. IEEE Transactions on Magnetics.

[CR68] Thielscher A, Pessoa L (2007). Neural correlates of perceptual choice and decision making during fear-disgust discrimination. The Journal of Neuroscience.

[CR69] Touroutoglou A, Hollenbeck M, Dickerson BC, Feldman BL (2012). Dissociable large-scale networks anchored in the right anterior insula subserve affective experience and attention. Neuroimage.

[CR70] Trautmann SA, Fehr T, Herrmann M (2009). Emotions in motion: Dynamic compared to static facial expressions of disgust and happiness reveal more widespread emotion-specific activations. Brain Research.

[CR71] Trautmann-Lengsfeld SA, Domínguez-Borràs J, Escera C, Herrmann M, Fehr T (2013). The Perception of Dynamic and Static Facial Expressions of Happiness and Disgust Investigated by ERPs and fMRI Constrained Source Analysis. PLoS One.

[CR72] Uddin LQ, Nomi JS, Hébert-Seropian B, Ghaziri J, Boucher O (2017). Structure and Function of the Human Insula. Journal of Clinical Neurophysiology.

[CR73] Verdade A, Castelhano J, Sousa T, Castelo-Branco M (2020). How positive emotional content overrules perceptual history effects: Hysteresis in emotion recognition. Journal of Vision.

[CR74] Wang Y, Metoki A, Smith DV, Medaglia JD, Zang Y, Benear S, Popal H, Lin Y, Olson IR (2020). Multimodal mapping of the face connectome. Nature Human Behaviour.

[CR75] Warburg E (1881). Magnetische untersuchungen [Magnetic investigations]. Annalen der Physik.

[CR76] Webster MA, Kaping D, Mizukami Y, Duhamel P (2004). Adaptation to natural facial categories. Nature.

[CR77] Wicker B, Keysers C, Plailly J, Royet JP, Gallese V, Rizzolatti G (2003). Both of us disgusted in My insula: The common neural basis of seeing and feeling disgust. Neuron.

[CR78] Williams D, Phillips G, Sekuler R (1986). Hysteresis in the perception of motion direction as evidence for neural cooperativity. Nature.

[CR79] Witthoft N, Sha L, Winawer J, Kiani R (2018). Sensory and decision-making processes underlying perceptual adaptation. Journal of Vision.

[CR80] Xue G, Lu Z, Levin IP, Bechara A (2010). The impact of prior risk experiences on subsequent risky decision-making: The role of the insula. Neuroimage.

[CR81] Zhang, Y., Zhou, W., Wang, S., Zhou, Q., Wang, H., Zhang, B., Huang, J., Hong, B., & Wang, X. (2018). The roles of subdivisions of human insula in emotion perception and auditory processing. *Cerebral Cortex,**29*(2), 1–12. 10.1093/cercor/bhx334 29342237

