# Supplementary material for: Positive hysteresis in emotion recognition: Face processing visual regions are involved in perceptual persistence, which mediates interactions between anterior insula and medial prefrontal cortex
Source: Cogn Affect Behav Neurosci. 2022 Jul 20;22(6):1275–89. doi: 10.3758/s13415-022-01024-w (PMC9622546; doi:10.3758/s13415-022-01024-w)
Supplement: Supplementary file 1 — (DOCX 18.7 kb) [file 13415_2022_1024_MOESM1_ESM.docx]

SUPPLEMENTARY DATA

**Supplementary Table 1 –** Summary of the selected brain regions of interest (ROIs) included in our study. Mean peak voxel MNI coordinates and number of voxels of bilateral Fusiform Face Area (FFA), Superior Temporal Sulcus (STS), and anterior Insula are described along with standard deviation (SD). ROIs were defined by contrasting the brain responses to visualization of static face images and rest (fixation cross) using an FFX-GLM analysis with a statistical threshold of q(FDR) = 0.05.

| ROI | X ± SD | Y ± SD | Z ± SD | # Voxels ± SD |
| --- | --- | --- | --- | --- |
| Right FFA | 41.28±2.10 | -51.99±2.97 | 19.29±2.56 | 568.41±90.28 |
| Left FFA | 39.24±3.79 | -52.14±4.07 | 18.39±2.79 | 460.18±200.65 |
| Right STS | 54.42±5.39 | -48.03±4.99 | 10.94±2.85 | 337.53±144.97 |
| Left STS | 49.50±5.33 | -48.26±6.16 | 11.82±7.26 | 274.76±169.11 |
| Right Anterior Insula | 35.69±4.17 | 18.99±8.44 | 2.75±5.88 | 213.24±147.28 |
| Left Anterior Insula | 34.35±2.59 | 15.99±11.03 | 6.23±3.57 | 297.65±111.87 |
